# Morphology-driven oxygen evolution performance of NiO_*x*_ nanostructures and implications for hole transport in perovskite solar cells

**DOI:** 10.1039/d6ra00607h

**Published:** 2026-02-18

**Authors:** Prabhu Bharathan, Can Li, Bipin Rijal, Lihua Zhang, Areesha Maryam, Joseph Delgado, Kim Kisslinger, Adyasa Priyadarsini, Mahesh Nepal, Tanka P. Bhushal, Tara P. Dhakal, Shyam Kattel, Jiye Fang

**Affiliations:** a Materials Science and Engineering Program, State University of New York at Binghamton Binghamton New York 13902 USA tdhakal@binghamton.edu jfang@binghamton.edu; b Department of Chemistry, State University of New York at Binghamton Binghamton New York 13902 USA; c Department of Electrical and Computer Engineering, Center for Autonomous Solar Power (CASP), Binghamton University Binghamton New York 13902 USA; d Center for Functional Nanomaterials, Brookhaven National Laboratory Upton New York 11973 USA; e Department of Physics, Florida A&M University Tallahassee Florida 32307 USA; f Department of Physics, University of Central Florida Orlando Florida 32816 USA shyam.kattel@ucf.edu

## Abstract

Morphology-controlled nanostructures provide an effective strategy to modulate both oxygen evolution reaction (OER) activity and photovoltaic performance in perovskite solar cells (PSCs). However, achieving low OER overpotentials and high power conversion efficiency (PCE) simultaneously through morphology engineering remains challenging. In this work, nickel oxide (NiO_*x*_) nanostructures with spindle-like (NiO_*x*_-NS) and plate-like (NiO_*x*_-NP) morphologies were synthesized and evaluated as bi-functional OER catalysts and hole transport layers (HTLs) in inverted PSCs. Structural and thermal analyses reveal that NiO_*x*_-NS crystallizes into a cubic phase at a lower temperature (300 °C), whereas NiO_*x*_-NP requires higher calcination temperatures, reflecting differences in precursor microstructure. Electrochemical measurements indicate that NiO_*x*_-NS calcined at 300 °C delivers the lowest OER overpotential (395 mV at 10 mA cm^−2^), outperforming NiO_*x*_-NP calcined at 400 °C (565 mV) and 500 °C (474 mV). This enhanced activity is ascribed to favorable surface strain, increased defect density, and advantageous facet exposure. When used as HTLs, NiO_*x*_-NS also delivers the highest PCE (13.25%) among all tested devices, exceeding those based on NiO_*x*_-NP and commercial NiO_*x*_, owing to improved hole extraction and interfacial contact. Overall, this study highlights the importance of morphology control and thermal processing in tailoring NiO_*x*_ for multifunctional nanomaterials in electrocatalytic and photovoltaic applications.

## Abstract 2

Morphology-controlled nanostructures provide an effective strategy to modulate both oxygen evolution reaction (OER) activity and photovoltaic performance in perovskite solar cells (PSCs).

## Graphical abstract

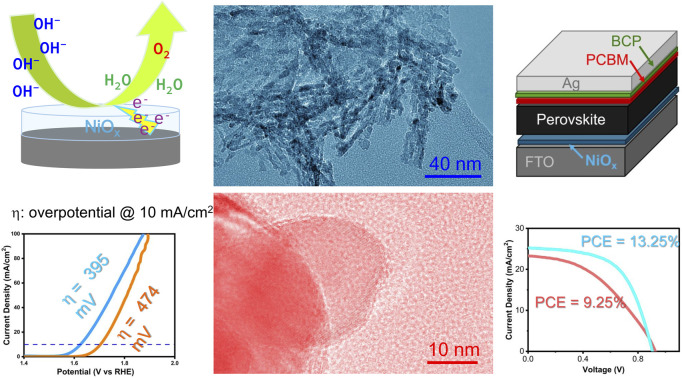

## 1. Introduction

Hydrogen production *via* electrochemical water splitting is a key technology for achieving a sustainable and carbon-neutral energy system.^1–3^ Among the two half-reactions, the oxygen evolution reaction (OER) in alkaline media is kinetically more demanding than the hydrogen evolution reaction (HER) due to its multistep, four-electron transfer pathway. This intrinsic complexity leads to sluggish reaction kinetics and high overpotentials.^4^ Consequently, the development of efficient, earth-abundant OER catalysts is essential for improving the overall efficiency of water electrolysis.

Nickel-based oxides (NiO_*x*_) have emerged as promising OER electrocatalysts owing to their low cost, chemical stability in alkaline media, and favorable electrochemical properties.^5–7^ However, despite extensive efforts, establishing a clear correlation between catalyst morphology and electrochemical performance remains challenging. Such understanding is critical, as catalyst morphology directly affects active-site exposure, charge-transfer characteristics, and reaction kinetics, which collectively govern catalytic efficiency.^8^ Elucidating morphology-performance relationships is therefore essential for the rational design of next-generation OER catalysts.

Beyond electrocatalysis, NiO_*x*_ is a p-type wide-bandgap semiconductor (>3.2 eV) that is widely used as a hole transport layer (HTL) in transparent conducting films,^9,10^ making it attractive for perovskite-based photovoltaic (PV) applications.^11,12^ Recent studies have demonstrated that bi-layered NiO_*x*_ architectures, comprising compact and nanoporous layers, can significantly enhance interfacial contact and reduce defect density, achieving a power conversion efficiency (PCE) of ∼20.7%.^13^ In addition, integration of NiO_*x*_ with self-assembled monolayers (SAMs) has been shown to improve interfacial properties, optimize energy-level alignment, and suppress defect formation in perovskite film.^14^ Furthermore, morphology-tailored NiO_*x*_ layers, particularly when combined with organic interlayers, effectively reduce open-circuit voltage losses in perovskite solar cells (PSCs).^15,16^ Notably, in both OER catalysis and HTL applications, NiO_*x*_ morphology plays a decisive role in determining functional performance.^17,18^

In this study, we systematically investigate the influence of NiO_*x*_ morphology on OER activity and its effectiveness as an HTL in PSCs. Two representative NiO_*x*_ nanostructures with distinct morphologies, spindle-like (NS) and plate-like (NP) nanostructures, were synthesized *via* controlled hydrothermal and thermal treatments, enabling direct comparison of their morphology-dependent structural, electrochemical, and PV properties. By correlating morphology with crystallization behavior, surface characteristics, and device performance, this work provides mechanistic insights into how morphology governs the multifunctional performance of NiO*_x_* nanomaterials.

## 2. Experimental section and computational method

### 2.1 Chemicals and materials

Nickel(ii) nitrate hexahydrate (Ni(NO_3_)_2_·6H_2_O, 99.99%), nickel(ii) acetate tetrahydrate (Ni(CH_3_CO_2_)_2_·4H_2_O, 98%), dimethylformamide (DMF, 99.8%), dimethyl sulfoxide (DMSO, 99%), isopropanol (IPA, 99.5%), and cetyltrimethylammonium bromide (CTAB, 95%) were purchased from Sigma-Aldrich. Methylammonium bromide (MABr, >99.99%), methylammonium iodide (MAI, >99.99%), formamidinium iodide (FAI, 99.99%), and methylammonium chloride (MACl, >99.99%) were obtained from Great Cell Solar Materials. Potassium hydroxide (KOH, 99.98%), urea (CO(NH_2_)_2_, > 99.9%), and bathocuproine (BCP, 98%) were supplied by Thermo Fisher Scientitic. Cesium iodide (CsI, 99.999%), lead iodide (PbI_2_, >99%), fullerene (C60, 99.95%), silver (Ag, 99.99%), and anhydrous ethanol (Koptec, 200 proof) were received from Acros Organics, Tokyo Chemical Industries (TCI), Solaris Chem, Kurt J. Lesker, and Decon Labs, Inc., respectively. All the chemicals were used as received without further purification. Ketjenblack EC600JD was provided by Lion Specialty Chemicals Co., Ltd (JP), and commercial nickel oxide (NiO_*x*_-com) nanoparticles were provided by US Research Nanomaterials Inc. (99.98%). Ultrapure de-ionized water with a resistivity of 18.2 MΩ·cm was obtained from a Purelab Flex3 water purification system (ELGA, UK).

### 2.2 Synthesis of NiO_*x*_ nanostructures

**Scheme 1:**
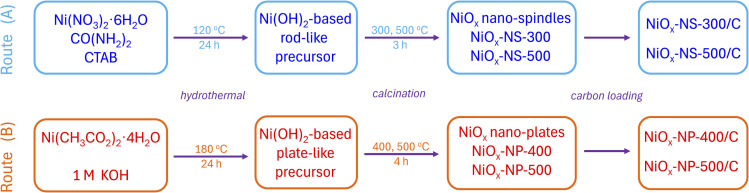
Schematic illustration of the synthesis routes for generating NiO_*x*_ nanostructures. (A) NiO_*x*_ nano-spindles (NiO_*x*_-NS) and (B) NiO_*x*_ nano-plates (NiO_*x*_-NP).

NiO_*x*_ nanocrystals with NS and NP morphologies were synthesized *via* hydrothermal and annealing routes by controlling the nickel precursors, pH regulation, and the use of CTAB,^19–21^ as illustrated in Scheme 1. NiO_*x*_-NS was obtained through urea-assisted hydrolysis combined with surfactant-directed anisotropic growth, whereas NiO_*x*_-NP was formed *via* direct KOH precipitation under strongly alkaline conditions using KOH. In the urea-assisted route (Route A), gradual pH evolution during hydrothermal treatment promotes the formation of hydroxide-based precursors, while CTAB, known to selectively adsorb on specific crystal facets and to stabilize surfaces with compatible surface energies,^19–21^ acts as a structure-directing agent, promoting anisotropic growth.^7^ In contrast, direct pH control with KOH (Route B) enables rapid precipitation of nickel hydroxide precursors, leading to the formation of NiO_*x*_-NP nanocrystals.

#### 2.2.1 Details of NiO_*x*_ nano-spindle synthesis and catalyst preparation

NiO_*x*_-NS samples were synthesized *via* a modified hydrothermal method adapted from previous work.^22^ In brief, 1.450 g of Ni(NO_3_)_2_·6H_2_O, 0.600 g of urea, and 0.911 g of CTAB were dissolved in 200 mL of ultrapure water under continuous stirring. The solution was transferred to a Teflon-lined stainless-steel autoclave and heated at 120 °C for 24 h. After naturally cooling to room temperature, the resulting precipitate was separated and washed three times using a 1 : 1 (v/v) mixture of ultrapure water and ethanol, followed by centrifugation. The collected solids were dried in a vacuum oven at 60 °C for 24 h and subsequently calcined in air at 300 °C or higher temperatures for 3 h. This process yielded NiO_*x*_ with a well-defined nano-spindle morphology.

A measured amount of NiO_*x*_-NS was ultrasonically dispersed in 5 mL of hexane and then added to a pre-sonicated suspension of carbon black (Ketjenblack EC600JD) in ethanol. The formulation was adjusted to achieve an estimated NiO_*x*_ loading of 60 wt% on carbon. The combined suspension underwent an additional 2 h of sonication and was left to settle overnight. The mixture was then centrifuged to remove the supernatant, and the resulting solid was air-dried. The final product, consisting of carbon-supported NiO_*x*_ nano-spindles, is denoted as NiO_*x*_-NS/C. For example, NiO_*x*_-NS-300/C and NiO_*x*_-NS-500/C refer to NiO_*x*_ nano-spindles on carbon calcined at 300 °C and 500 °C for 3 h, respectively.

#### 2.2.2 Details of NiO_*x*_ nanoplate synthesis and catalyst preparation

NiO_*x*_-NP samples were synthesized *via* a two-step process. First, Ni(OH)_2_ nanoplates were prepared using a hydrothermal method adapted from the literature.^23^ Specifically, 0.107 g of Ni(CH_3_CO_2_)_2_·4H_2_O was dissolved in 50 mL of ultrapure water, followed by the addition of 1 mL of 1 M KOH to raise the pH to ∼13. The solution was transferred to a Teflon-lined autoclave and heated at 180 °C for 24 h. After cooling to room temperature, the supernatant was removed, and the precipitate was washed three times with a 3 : 1 (v/v) mixture of ultrapure water and ethanol. The product was collected by centrifugation and air-dried. In the second step, the resulting Ni(OH)_2_ nanoplates were calcined in air at 400 °C and 500 °C for 4 h in a tube furnace, producing NiO_*x*_ nanoplates labeled as NiO_*x*_-NP-400 and NiO_*x*_-NP-500, respectively.

Each of the NiO_*x*_-NP sample was then loaded on Ketjenblack EC600JD by following the same procedure used for NiO_*x*_-NS/C. The resulting carbon-supported materials are designated as NiO_*x*_-NP-400/C and NiO_*x*_-NP-500/C, corresponding to their respective calcination temperatures.

### 2.3 Working electrode preparation and electrochemical measurements

To prepare the catalyst ink, 5 mg of NiO nanocrystals were dispersed in a mixture containing 1.0 mL of acetone and 20.0 µL of 5% Nafion® solution, followed by ultrasonication for 1 h to achieve uniform dispersion. Subsequently, 30.0 µL of the ink was drop-cast onto a pre-cleaned glassy carbon (GC) rotating disk electrode (RDE) (5 mm diameter; Pine Research Instrumentation) and allowed to dry at room temperature.

Electrochemical measurements were conducted at room temperature on a Gamry 1000 E workstation using a standard three-electrode setup. The working electrode was a GC RDE coated with the carbon-supported catalyst, while a saturated Ag/AgCl (4 M KCl) electrode and a graphite rod served as the reference and counter electrodes, respectively. All potentials were converted to the reversible hydrogen electrode (RHE) scale using *E*_RHE_ = *E*_Ag/AgCl_ + 1.0258 (*V*), and the OER overpotential was calculated as *E*_OER_ = *E*_RHE_ − 1.23(*V*).

### 2.4 Perovskite precursor solution preparation

A 1.5 M Cs_0.05_FA_0.84_MA_0.11_Pb(I_0.985_Br_0.015_)_3_ perovskite precursor solution was prepared by mixing 6.55 mg of MABr, 16.93 mg of MAI, 19.49 mg of CsI, 216.68 mg of FAI, 746.8 mg of PbI_2,_ and 8.1 mg of MACl in 1 mL of DMF : DMSO (4 : 1 v/v) solvent mixture. The solution included 8% excess PbI_2_ and MACl to promote improved perovskite crystallization and film formation. After stirring overnight, the solution was filtered using a 0.22 µm PTFE syringe filter before use.

### 2.5 Fabrication of perovskite solar cells

Fluorine-doped tin oxide (FTO) coated glass substrates (2.5 × 2.5 cm^2^) were washed with detergent and deionized water before being sonicated for 5 min each in acetone and IPA. The substrates were then dried using a nitrogen stream and treated with UV-ozone for 30 min. For PCE assessment, NiO_*x*_ inks were prepared by dispersing 15 mg of the respective NiO_*x*_ nanomaterials in 1 mL of a 1 : 1 (v/v) IPA: ethanol solvent mixture. These inks were statically spin-coated onto the cleaned FTO substrates at 4000 rpm for 30 s, followed by thermal annealing on a hot plate at 140 °C for 10 min under ambient conditions. Subsequent device fabrication was performed inside a nitrogen-filled glovebox maintained at <1 ppm O_2_ and <5 ppm H_2_O. The perovskite layer was deposited using a two-step spin-coating process. The precursor solution was statically dispensed and spin-coated at 1000 rpm for 10 s, followed by 5000 rpm for 25 s. During the final 10 s of the second spin step, 300 µL of chlorobenzene was dripped onto the rotating substrate to induce rapid crystallization. The resulting film was immediately annealed on a hot plate at 100 °C for 1 h, yielding a uniform, black, mirror-like perovskite film. A 15 nm layer of C_60_ was then deposited *via* thermal evaporation as the electron transport layer. This was followed by dynamic spin-coating of a 5 nm BCP layer (0.5 mg mL^−1^ in IPA) at 5000 rpm for 20 s and subsequent annealing at 80 °C for 5 min. Finally, an 80 nm Ag top electrode was thermally evaporated under ultrahigh vacuum (∼10^−7^ Torr), completing the device architecture.

### 2.6 Current–voltage characterization of the solar cell

Current–voltage (*J*–*V*) measurements were performed using a Keithley 4200-SCS semiconductor characterization system in conjunction with a solar simulator (Photo Emission Tech) under standard AM1.5 G illumination at 100 mW cm^−2^. The voltage was swept in the reverse direction at a scan rate of 0.01 V s^−1^. The active area of each tested device was 0.09 cm^2^.

### 2.7 Other characterization methods

X-ray diffraction (XRD) patterns were recorded using a PANalytical X'pert Pro diffractometer equipped with a Cu K_α1_ radiation source. Transmission electron microscopy (TEM) was performed on a JEOL 2100 F microscope operated at 200 kV. For TEM characterization, nanocrystal samples (without carbon support) were dispersed in hexane and drop-cast onto TEM grids, followed by cleaning to remove residual organics. Thermogravimetric analysis (TGA) was carried out under a nitrogen atmosphere using a TA Instruments Q50 system (TA-Q50 Thermo Balance), with samples heated from room temperature to 500 °C at 10 °C min^−1^. Raman spectra were acquired using a DXR Raman microscope (Thermo Scientific) equipped with a 532 nm excitation laser, operated at a power of 2 mW. High-resolution TEM (HRTEM) was performed on Thermo Fisher Talos 200X.

### 2.8 Computational methods

All the spin-polarized Density Functional Theory (DFT) calculations were performed using the Vienna *Ab initio* Simulation Package (VASP) code.^24^ The projector augmented wave (PAW)^25^ method was used to describe the interaction between the frozen core and free valence electrons. Perdew–Burke–Ernzerhof (PBE) exchange–correlation function,^26^ within generalized gradient approximation (GGA) calculations, along with semiempirical D3 dispersion correction, for van der Waal's interaction, was used for all the calculations.^27,28^ The cutoff energy of structure optimization was set to 450 eV throughout the simulation. A vacuum of 18 Å was added along the direction normal to the catalyst surface to avoid the error due to self-interaction among periodic slabs. A Monkhorst–Pack grid of 5 × 5 × 1 *k*-point mesh was used to sample the Brillouin zone for NiO(001) surface. The structural relaxation was performed iteratively until the energy and force criteria converged to 10^−5^ eV and 10^−2^ eV Å^−1^. The Hubbard U parameter (*U*_eff_) of 6.00 eV is used for Ni on NiO.^29^

Considering the four-electron transfer pathway using OH^−^ (alkaline medium) as the active species, the four steps of the OER are described as

r1 * + OH^−^ → *OH + e^−^

r2 *OH + OH^−^ → *O + H_2_O_(l)_ + e^−^

r3 *O + OH^−^ → *OOH + e^−^

r4 *OOH + OH^−^ → * + O_2(g)_ + H_2_O_(l)_ + e^−^

where, * represents the active site on the catalyst.

Accordingly, the Gibbs free energy change (Δ*G*) of each elementary step is given by,1
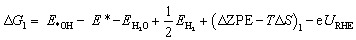
2
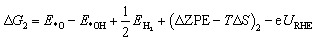
3
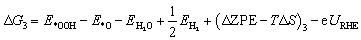
4
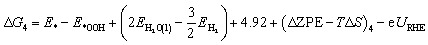
where, *E*_i_ = electronic energy (*i* = intermediates), ZPE_*i*_ = zero-point energy, and TS_*i*_ = entropic correction of the surface adsorbed species.

## 3. Results and discussion

### 3.1. Morphology-controlled synthesis and characterization of NiO_x_

Thermal transformation of hydroxide precursors with different morphologies was examined by TGA and derivative thermogravimetry (DTG), as shown in Fig. S1. The NiO_*x*_-NS precursor from Route (A) exhibited two major mass losses at ∼300 °C (∼13.72 wt%) and ∼340 °C (∼30.86 wt%) (Fig. S1a), attributed to CTAB-assisted conversion to NiO_*x*_ followed by formation of non-stoichiometric phases at higher temperature. In contrast, the NiO_*x*_-NP precursors from Route (B) began losing mass at ∼250 °C, indicating lower thermal stability, with a sharp loss between 275 and ∼300 °C marking the onset of NiO_*x*_-NP formation (Fig. S1b).

**Fig. 1:**
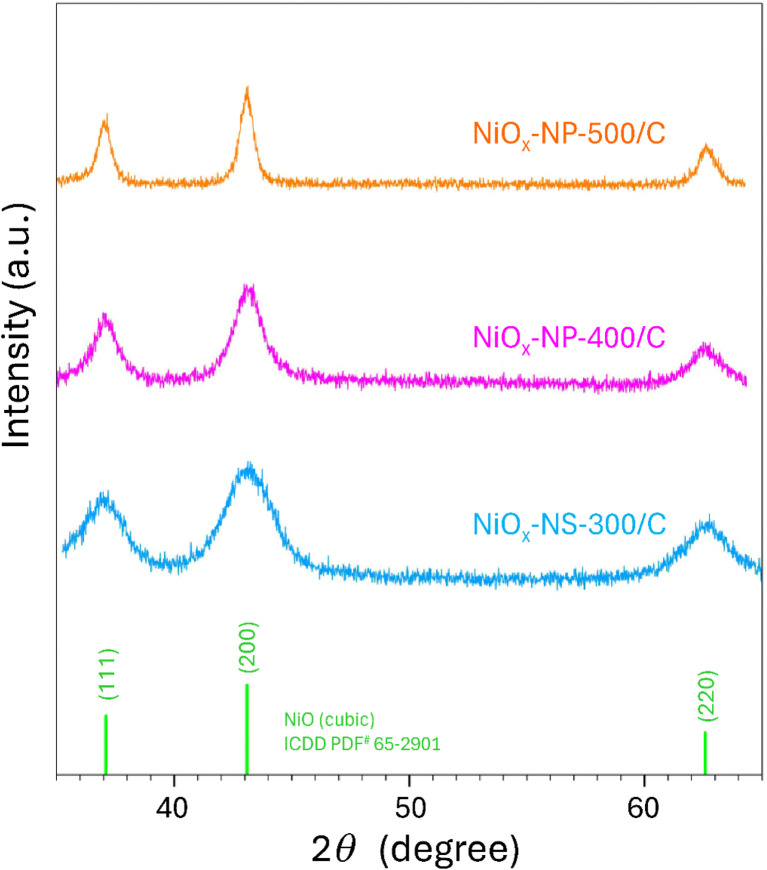
XRD patterns of carbon-supported NiO_*x*_-NS-300/C, NiO_*x*_-NP-400/C, NiO_*x*_-NP-500/C, along with the reference pattern for cubic NiO (ICDD PDF 65-2901).

The crystallinity of carbon-supported NiO_*x*_ samples calcined at different temperatures was further examined by XRD (Fig. 1). NiO_*x*_-NS fully crystallized at 300 °C after 3 h calcination (NiO_*x*_-NS-300/C), whereas NiO_*x*_-NP required ≥400 °C for 4 h to achieve complete crystallization. Clear NiO diffraction peaks were observed for NiO_*x*_-NS-300/C, while NP precursors treated at 300 °C retained the hexagonal Ni(OH)_2_ phase with no detectable NiO reflections (XRD pattern not shown). Complete phase transformation was achieved for carbon-supported NP samples calcined for 4 h at 400 °C (NiO_*x*_-NP-400/C) and 500 °C (NiO_*x*_-NP-500/C), both showing well-defined cubic NiO diffraction patterns (ICDD PDF 65-2901). The rhombohedral NiO phase is excluded by the absence of characteristic peak splitting at 2*θ* = 37.24–37.30° and 62.84–62.92° (ICDD PDF 89-3080), consistent with HRTEM (*vide infra*). These results indicate that NiO_*x*_-NS crystallizes fully at a lower temperature, whereas NiO_*x*_-NP requires higher calcination temperatures, with all final products adopting the cubic phase.

**Fig. 2:**
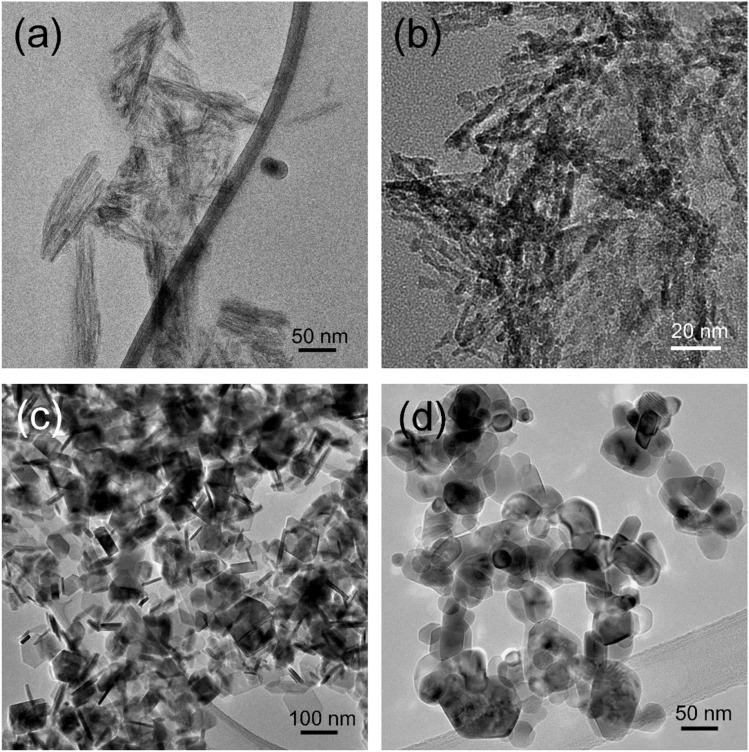
TEM images of Ni(OH)_2_ precursors and the resulting NiO_*x*_ nanostructures. (a) As-synthesized Ni(OH)_2_-based nano-spindle precursor (Route A); (b) NiO_*x*_ nano-spindles obtained by annealing the Ni(OH)_2_ nano-spindles at 300 °C for 3 h (*e.g.*, NiO_*x*_-NS-300); (c) As-synthesized Ni(OH)_2_-based nano-plate precursor (Route B); and (d) NiO_*x*_ nano-plates obtained by annealing the Ni(OH)_2_ nano-plates at 500 °C for 4 h (*e.g.*, NiO_*x*_-NP-500).

**Fig. 3:**
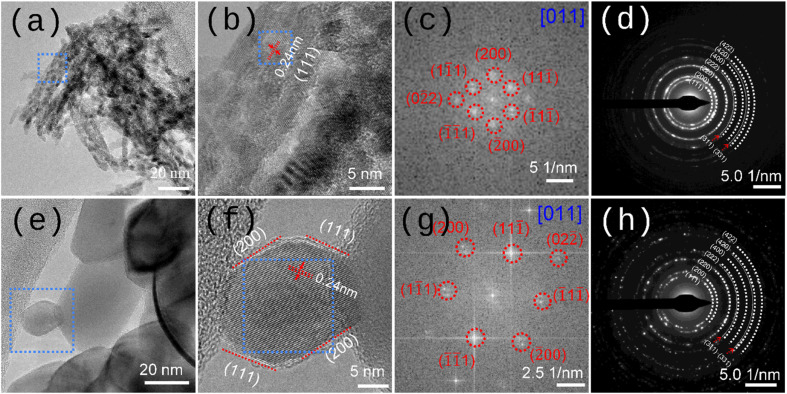
High-resolution TEM (HRTEM) analysis and selected area electron diffraction (SAED) patterns. (a–d), HRTEM image of pristine NiO_*x*_-NS-300: (a) HRTEM image; (b) zoomed-in view of the region marked by the blue box, showing measured lattice spacing; (c) corresponding diffractogram of the zoomed-in area; (d) SAED pattern. (e–h), Corresponding HRTEM observations for pristine NiO_*x*_-NP-500: (e), overview image; (f) zoomed-in view with lattice spacing; (g) diffractogram; (h) SAED pattern.

Fig. 2 compares the morphologies of the hydroxide precursors obtained from both synthetic routes and their corresponding free-standing NiO_*x*_ nanostructures. Route (A) yields clustered nanowires (∼25 nm in diameter and 80–100 nm in length, Fig. 2a), which transform into nano-spindles (NiO_*x*_-NS-300) after calcination at 300 °C for 3 h (Fig. 2b). Each NS consists of multiple polycrystalline NiO_*x*_ domains composed of 5–7 nm primary crystallites (also see Fig. 3a), in good agreement with the crystalline size of 5.6 nm estimated from the Scherrer equation^30^ based on (200) peak broadening (1.5114°) in Fig. 1. In contrast, Route (B) produces monodisperse hexagonal platelets with well-defined edges, ∼60 nm in lateral size and <8 nm in thickness (Fig. 2c). Subsequent calcination at 500 °C for 4 h (NiO_*x*_-NP-500) results in a mixture of large- and small-area NiO_*x*_ nanoplates with reduced thickness (Fig. 2d).

HRTEM was employed to examine the crystallographic structure of pristine NiO_*x*_ samples prepared without carbon support. For NiO_*x*_-NS-300, well-defined lattice fringes are observed (Fig. 3a and b), with an interplanar spacing of ∼0.24 nm derived from the fast Fourier transform (FFT) diffractogram (Fig. 3c), consistent with the (111) lattice spacing of cubic NiO (*d*_111_ = 2.4217 Å, ICDD PDF 65-2901). The corresponding selected area electron diffraction (SAED) pattern (Fig. 3d) confirms a single-phase cubic structure. A similar analysis of NiO_*x*_-NP-500 (Fig. 3e–h) reveals pronounced lattice fringes, indicative of high crystallinity. The magnified region (Fig. 3f) shows an interplanar spacing of ∼0.24 nm corresponding to the {111} planes of cubic NiO, with the nanoplate oriented along the [011] zone axis. This suggests that the exposed edges of NiO_*x*_-NP-500 correspond to {111} and {200} facets (Fig. 3f), and the basal surface of the nanoplate is most likely parallel to the {110} facet. This structural assignment is supported by the FFT diffractogram (Fig. 3g), which resolves the {111} reflections, and by the SAED pattern (Fig. 3h), which exhibits sharp diffraction spots characteristic of single-phase cubic NiO. The consistent lattice spacings and diffraction features observed for both NiO_*x*_-NS-300 and NiO_*x*_-NP-500 confirm their high crystallinity and a cubic phase, regardless of synthesis route. Nevertheless, the distinct morphologies and local structure features indicate synthesis-dependent microstructural variations that may influence physical properties. The distinct NiO_*x*_ crystallization observed for the two types of precursors (Fig. 1 and S1) can be attributed to differences in precursor domain size and NiO_*x*_ evolution pathways. Specifically, NS precursors consist of thin nanowires (∼25 nm in diameter) that fragment into discrete small nanocrystals during conversion to NiO_*x*_, whereas NP precursors comprise larger platelets (∼25 nm in lateral size) and undergo comparatively limited structural reorganization. The higher surface energy associated with the NS precursors consequently lowers the thermal energy barrier for phase transformation, enabling NiO_*x*_ crystallization at lower calcination temperatures than required for NP precursors.

Raman spectroscopy was used to probe optical phonon modes and defects in both NiO_*x*_-NS-300 and NiO_*x*_-NP-500 (Fig. S2). The NiO_*x*_-NS-300 spectrum (Fig. S2a) shows peaks at 345–355, 520–530, 740–750, and 1070–1080 cm^−1^, corresponding to the one-phonon transverse optical (TO), one-phonon longitudinal optical (LO), second-order transverse optical (2TO), and second-order longitudinal optical (2LO) modes, respectively,^31–34^ consistent with NiO nanorods (30–80 nm) previously reported.^35^ Defect-induced lattice symmetry breaking activates otherwise forbidden modes. Accordingly, a feature near 250 cm^−1^ within the one-phonon band is attributed to defect-induced phonons associated with lattice distortion and vacancies.^36–38^ In contrast, NiO_*x*_-NP-500 (Fig. S2b) exhibits only two dominant peaks at 490–500 and 1060–1070 cm^−1^, assigned to the LO^36^ and 2LO^39^ modes, respectively. The absence of TO and defect-related modes indicates a lower defect concentration in nano-plates, likely arising from their planar morphology and implying distinct material properties relative to nano-spindles.

### 3.2. OER performance

**Fig. 4:**
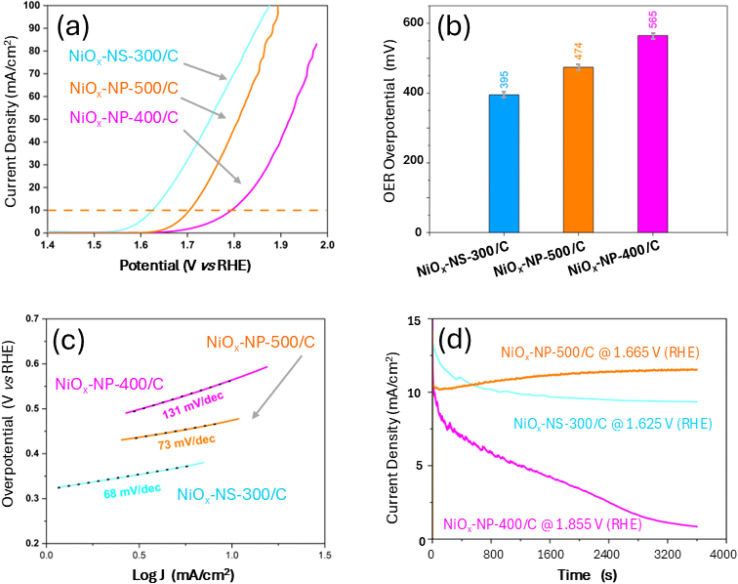
OER performance of NiO_*x*_-NS-300/C, NiO_*x*_-NP-400/C, and NiO_*x*_-NP-500/C. (a) Polarization curves (current density *vs.* potential) recorded in 1 M KOH at an electrode rotation speed of 1600 rpm. The horizontal dashed line indicates the benchmark current density of 10 mA cm^−2^, used to evaluate the OER overpotential; (b) OER overpotentials of three catalysts at a current density of 10 mA cm^−2^ 1 M KOH; (c) Tafel slopes of three catalysts obtained from (a–d) chronoamperometry plots showing current density *vs.* time for NiO_*x*_-NS-300/C (at 1.625 V *vs.* RHE), NiO_*x*_-NP-400/C (at 1.855 V *vs.* RHE), and NiO_*x*_-NP-300/C (at 1.665 V *vs.* RHE), measured in 1 M KOH at a rotation speed of 1600 rpm using an RDE for a duration of 1 h.

OER polarization curves of the NiO_*x*_ catalysts, measured in 1 M KOH using a three-electrode configuration with a GC RDE, are shown in Fig. 4a. At 10 mA cm^−2^, NiO_*x*_-NS-300/C, NiO_*x*_-NP-400/C, and NiO_*x*_-NP-500/C exhibited overpotentials of 395 mV, 565 mV, and 474 mV *vs.* RHE, respectively (Fig. 4b). Among nanoplate catalysts, NiO_*x*_-NP-500/C outperformed NiO_*x*_-NP-400/C, indicating enhanced OER activity with higher calcination temperature.

In alkaline media, OER proceeds *via* a four-electron transfer mechanism,^40^ with overpotential largely governed by OH^−^ adsorption.^4^ The lower overpotential of NiO_*x*_-NS-300/C indicates weaker OH^−^ adsorption (Fig. 4a),^41–43^ which contributes to its superior activity. As evidenced in Fig. 3 and S2, this behavior is attributed to the tensile strain induced by oxygen vacancies^44^ and inter-crystal defects,^45^ as well as exposure of high-index facets that increase active sites^46^ and promote favorable OH^−^ adsorption–desorption kinetics,^47^ consistent with its low Tafel slope of 68 mV dec^−1^ (Fig. 4c). The spindle morphology further enhances accessible surface area and charge/mass transport.^48,49^ In contrast, NiO_*x*_-NP predominantly exposes low-index facets (Fig. 3f), such as {111} surfaces, which are known to bind intermediates more strongly and limit activity.^50^ The fraction of more active {001} facets is therefore significantly lower in NiO_*x*_-NP than in NiO_*x*_-NS-300, explaining the higher overpotentials of the nanoplate catalysts.

**Fig. 5:**
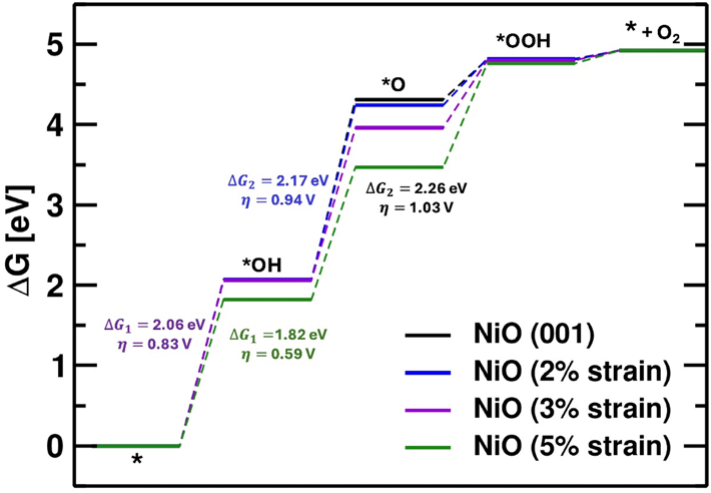
Free energy diagram of OER on NiO(001), calculated using first-principles Density Functional Theory (DFT) method.

Despite their similar morphologies, NiO_*x*_-NP-400/C and NiO_*x*_-NP-500/C exhibit markedly different OER performance. NiO_*x*_-NP-400/C shows a higher overpotential and larger Tafel slope (565 mV at 10 mA cm^−2^, 131 mV dec^−1^) than NiO_*x*_-NP-500/C (474 mV, 73 mV dec^−1^, Fig. 4b and c), indicating stronger OH^−^ adsorption and slower kinetics. DFT calculations on NiO(001) surfaces (Fig. 5) reveal that tensile strain can reduce the theoretical overpotential from 1.03 V (unstrained) to 0.59 V at 5% strain, indicating a strain-assisted lowering of the rate-limiting barrier. Experimentally, however, NiO_*x*_-NP-500/C, which is expected to have less tensile strain, exhibits a lower overpotential than NiO_*x*_-NP-400/C. This discrepancy suggests that no strain exists and/or the dominant exposed facets in NiO_*x*_-NP may not be the highly active {001} facets,^50^ but rather less active facets such as {011}, consistent with HRTEM analysis. Interestingly, the NiO_*x*_-NS system shows lower overpotentials and relatively consistent behavior across calcination temperatures. At 10 mA cm^−2^, the OER overpotentials for NiO_*x*_-NS-300/C (395 mV) and NiO_*x*_-NS-500/C (364 mV) are very close (Fig. S3), with only a slight decrease for the higher-temperature sample, despite the theoretical prediction that NiO_*x*_-NS-300/C should exhibit a lower OER overpotential still not being observed. These results confirm that the spindle-like NiO_*x*_-NS morphology provides superior intrinsic activity and that its OER performance is largely insensitive to calcination temperature, highlighting its advantage for efficient water oxidation.

Chronoamperometry tests (Fig. 4d) conducted for 1 h at 1600 rpm in 1 M KOH, at 1.625 V (NiO_*x*_-NS-300/C), 1.855 V (NiO_*x*_-NP-400/C), and 1.665 V (NiO_*x*_-NP-500/C) *vs.* RHE, corresponding to their respective 10 mA cm^−2^ overpotentials, reveal distinct stability trends. NiO_*x*_-NP-500/C shows stable, gradually increasing current, indicating excellent durability and possible *in situ* activation, whereas NiO_*x*_-NP-400/C undergoes rapid current decay. NiO_*x*_-NS-300/C exhibits only a minor decline, indicating superior stability relative to NiO_*x*_-NP-400/C. These trends are attributed to electrochemically active surface area (ECSA) stabilization and degradation dynamics under forced convection, which enhances oxygen removal but may accelerate catalyst dissolution and ECSA loss. Although no visible dissolution was observed, further analyses are required to confirm Ni leaching. Overall, these results demonstrate that morphology and stoichiometry critically govern both OER activity and durability, underscoring the importance of coordinated structural design and thermal processing in Ni-based oxide electrocatalysts.

### 3.3. Photovoltaic performance

To further assess morphology-dependent NiO_*x*_ performance as HTLs, NiO_*x*_-NS-300 and NiO_*x*_-NP-500 were incorporated into PSCs, with a commercial NiO_*x*_ (NiO_*x*_-com) used as a reference. This comparison is intended to evaluate relative HTL performance under identical testing conditions, rather than to achieve the maximum absolute PCE. Consequently, the measurements were performed without exhaustive optimization, although state-of-the-art PSC architectures and processing were employed.

**Fig. 6:**
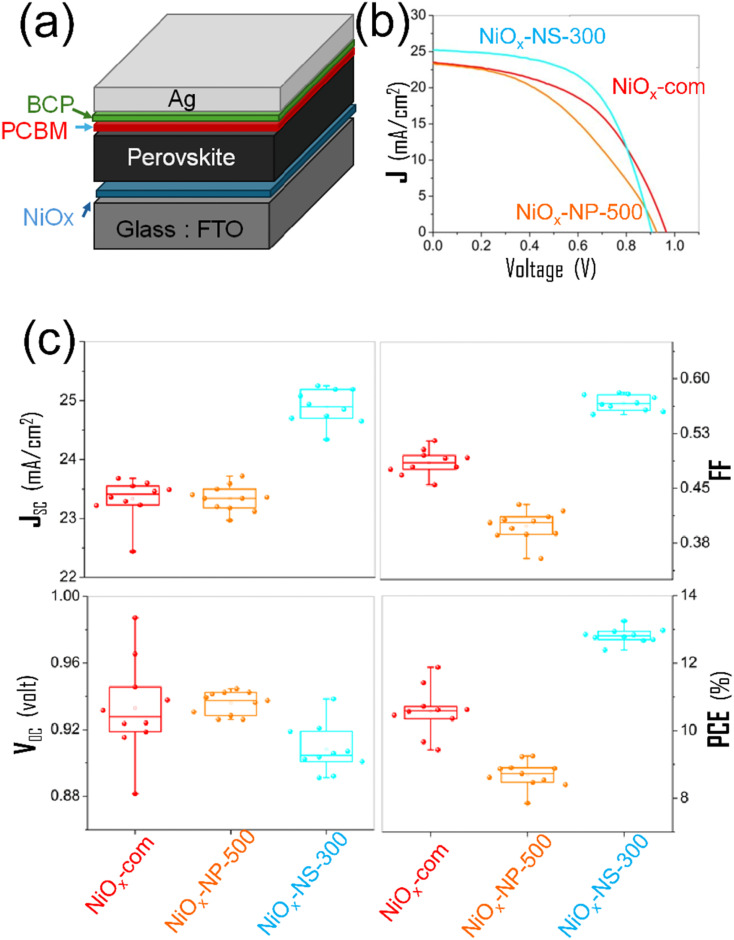
Photovoltaic (PV) performance of perovskite solar cells (PSCs) incorporating different NiO_*x*_ as hole transport layers (HTLs). (a) Schematic of the inverted PSC architecture using nanostructured NiO_*x*_ as the HTL; (b) *J*–*V* curves of the best-performing devices based on NiO_*x*_-NS-300, NiO_*x*_-NP-500, and commercial NiO_*x*_ (NiO_*x*_-com); (c) box-and-whisker plots, summarizing the short-circuit current density (*J*_sc_), open-circuit voltage (*V*_oc_), fill factor (*FF*), and power conversion efficiency (PCE) of devices with different NiO_*x*_-based HTLs.

Fig. 6 presents the device structure (Fig. 6a) and PV performance (Fig. 6b) of PSCs using different NiO_*x*_-based HTLs. *J*–*V* curves and box-and-whisker plots reveal that NiO_*x*_-NS-300 delivers the highest efficiency among the tested devices. As shown in Fig. 6b and c, the best NiO_*x*_-NS-300-based PSC achieved a PCE of 13.25%, with an open-circuit voltage (*V*_oc_) of 0.91 V, a short-circuit current density (*J*_sc_) of 25.19 mA cm^−2^, and a fill factor (*FF*) of 58.1%. In comparison, the best NiO_*x*_-NP-500 device reached a PCE of 9.25%, with *V*_oc_ = 0.93 V, *J*sc = 23.34 mA cm^−2^, and *FF* = 42.8%), while the NiO_*x*_-com device achieved 11.88% (*V*_oc_ = 0.98 V, *J*_sc_ = 23.36 mA cm^−2^, *FF* = 51.5%).

The superior performance of NiO_*x*_-NS-300 arises primarily from enhanced *J*_sc_ and *FF*, indicating more efficient hole extraction at the NiO_*x*_/perovskite interface, improved interfacial contact, favorable energy-level alignment, and higher bulk conductivity.^51,52^ Ink photographs (Fig. S4) show that NiO_*x*_-NS-300 exhibits a darker (black) coloration than NiO_*x*_-NP-500 and NiO_*x*_-com, suggesting a distinct Ni oxidation state^53^ associated with stronger p-type character and improved conductivity.^54^ Additionally, the narrower PCE distribution for NiO_*x*_-NS-300-based devices indicates improved HTL uniformity, enabling more consistent layer deposition and enhanced device reproducibility.

Although OER catalysis and hole transport in PSCs operate through distinct mechanisms and performance metrics, both functions are strongly governed by NiO_*x*_ morphology. In OER, spindle-like NiO_*x*_ benefits from defect-rich surfaces, favorable facet exposure, lattice strain effects, and enhanced charge-transport pathways, which collectively facilitate intermediate adsorption and accelerate reaction kinetics; whereas in PSCs, these same characteristics promote enhanced hole hopping transport and more efficient hole extraction at the NiO_*x*_/perovskite interface. This comparison highlights that morphology-induced structural features can simultaneously optimize NiO_*x*_ performance across electrochemical and photovoltaic applications.

## 4. Conclusions

In conclusion, NiO_*x*_ nanostructures with spindle- and plate-like morphologies were synthesized *via* controlled hydrothermal and annealing processes, enabling a systematic evaluation of morphology-dependent properties. The spindle-like NiO_*x*_, which crystallized at a lower temperature (300 °C), exhibited superior OER activity, delivering lower overpotentials (395 mV *vs.* 565 and 474 mV at 10 mA cm^−2^) and smaller Tafel slopes (68 mV dec^−1^*vs.* 131 and 73 mV dec^−1^) than NiO_*x*_ nanoplates calcined at 400 and 500 °C, respectively, together with enhanced operational stability. Beyond electrocatalysis, spindle-like NiO_*x*_ also demonstrated superior performance as a hole transport layer in perovskite solar cells, achieving higher device efficiency and improved uniformity compared with plate-like NiO_*x*_ and commercial NiO_*x*_ specimens. The best spindle-like NiO_*x*_-based device reached a power conversion efficiency of 13.25% (*V*_oc_ = 0.91 V, *J*_sc_ = 25.19 mA cm^−2^, *FF* = 58.1%), exceeding those using a plate-like NiO_*x*_-based device or the commercial benchmark. The distinct crystallization behaviors from the two types of precursors are attributed to differences in their microstructures, while the enhanced OER and photovoltaic performance of spindle-like NiO_*x*_ is ascribed to favorable facet exposure, tensile strain, higher defect density, and enhanced interfacial charge transport, as supported by structural characterization and DFT analysis. Overall, this work highlights morphology control and thermal optimization as effective strategies to tailor phase transformation, electrocatalytic activity, and charge-transport properties, providing guidance for the design of multifunctional NiO_*x*_ materials for water oxidation and perovskite photovoltaic applications.

## Author contributions

P. B., C. L., and B. R. performed synthesis and device fabrication. P. B., C. L., B. R., L. Z., A. M., K. K., M. N., and T.B. carried out characterization and data curation. A. P. and S. K. conducted computation and modeling. P. B., C. L., B. R., L. Z., J. D., and A. M. conducted formal analysis. T. D., S. K., and J. F. contributed to supervision, funding acquisition, and conceptualization. P. B., B. R., J. F., S. K., and T. D. prepared the original draft. All authors reviewed, edited, and approved the final manuscript.

## Conflicts of interest

There are no conflicts to declare.

## Supplementary Material

RA-016-D6RA00607H-s001

## Data Availability

All data associated with this study are available in the article and supplementary information (SI). Supplementary information: TGA/DTG profiles of both precursors, Raman spectra of pristine NiO_x_-NS-300 and NiO_x_-NP-500, polarization curves (recorded in 1 M KOH) for all samples used in OER evaluation, and photographs of inks prepared for PSC testing. See DOI: https://doi.org/10.1039/d6ra00607h.

## References

[cit1] Liu S., Wei Y., Wang M., Shen Y. (2025). Coord. Chem. Rev..

[cit2] Yu Z.-Y., Duan Y., Feng X.-Y., Yu X., Gao M.-R., Yu S.-H. (2021). Adv. Mater..

[cit3] Hu C., Zhang L., Gong J. (2019). Energy Environ. Sci..

[cit4] Suntivich J., May K. J., Gasteiger H. A., Goodenough J. B., Shao-Horn Y. (2011). Science.

[cit5] Nardi K. L., Yang N., Dickens C. F., Strickler A. L., Bent S. F. (2015). Adv. Energy Mater..

[cit6] Babar P. T., Lokhande A. C., Gang M. G., Pawar B. S., Pawar S. M., Kim J. H. (2018). J. Ind. Eng. Chem..

[cit7] Silva V. D., Simões T. A., Grilo J. P. F., Medeiros E. S., Macedo D. A. (2020). J. Mater. Sci..

[cit8] Li X., Ge L., Du Y., Huang H., Ha Y., Fu Z., Lu Y., Yang W., Wang X., Cheng Z. (2023). ACS Nano.

[cit9] Zhu A.-Z., Shan H., Cai S.-M., Chang C.-C., Yang L., Deng C.-H., Zhou N.-N., Hu K.-H., Yu H., Lv J.-G., He G. (2025). Rare Met..

[cit10] Wang Q., Liu H., Fan C., Tang P., Li B., Zhang L., Shi J. (2025). Adv. Funct. Mater..

[cit11] Marion D., Mounir M., Ivan M., Quentin J., Sylvain N., Christophe B., Adriana P. (2025). Mater. Sustain..

[cit12] Cao F., Dai X., Tian D., Peng Y., Yin J., Li J., Yang Y., Zheng N., Wu B. (2025). Energy Environ. Sci..

[cit13] Kuo D.-W., Chen C.-T. (2025). ACS Appl. Energy Mater..

[cit14] Guo Y., Huang L., Wang C., Huang J., Liu S., Liu X., Zhang J., Hu Z., Zhu Y. (2024). J. Mater. Chem. C.

[cit15] Zhao Z., Liu W., Kong T., Liu Y., Chen W., Gao P., Bi D. (2025). Adv. Funct. Mater..

[cit16] Mukherjee K., Kreugel D., Phung N., van Helvoirt C., Zardetto V., Creatore M. (2024). Mater. Adv..

[cit17] Barkaoui S., Wang Y., Zhang Y., Gu X., Li Z., Wang B., Baiker A., Li G., Zhao Z. (2024). iScience.

[cit18] Zhang H., Hou W., Deng Y., Song J., Zhang F. (2025). J. Mater. Chem.
A.

[cit19] Zhou M., Li C., Fang J. (2021). Chem. Rev..

[cit20] Li C., Luan Y., Zhao B., Kumbhar A., Zhang F., Fang J. (2020). MRS Adv..

[cit21] Li C., Pan J., Chen X., Zhang L., Dennett A., Bharathan P., Lee D., Zhou G., Fang J. (2024). Electron.

[cit22] Justin P., Meher S. K., Rao G. R. (2010). J. Phys. Chem. C.

[cit23] Dionigi F., Reier T., Pawolek Z., Gliech M., Strasser P. (2016). ChemSusChem.

[cit24] Kresse G., Furthmüller J. (1996). Phys. Rev. B: Condens. Matter.

[cit25] Blöchl P. E. (1994). Phys. Rev. B: Condens. Matter.

[cit26] Perdew J. P., Burke K., Ernzerhof M. (1996). Phys. Rev. Lett..

[cit27] Grimme S. (2004). J. Comput. Chem..

[cit28] Grimme S. (2006). J. Comput. Chem..

[cit29] Yun Y. H., Kim K., Lee C., An B.-S., Kwon J. H., Lee S., Kim M., Seo J., Park J. H., Kim B.-H., Cho H.-S. (2023). J. Energy Chem..

[cit30] Fang J., Stokes K. L., Wiemann J. A., Zhou W. L., Dai J., Chen F., O'Connor C. J. (2001). Mater. Sci. Eng., B.

[cit31] Mironova-Ulmane N., Kuzmin A., Sildos I., Pärs M. (2011). Cent. Eur. J. Phys..

[cit32] Sunny A., Balasubramanian K. (2020). J. Phys. Chem. C.

[cit33] Gandhi A. C., Pant J., Pandit S. D., Dalimbkar S. K., Chan T.-S., Cheng C.-L., Ma Y.-R., Wu S. Y. (2013). J. Phys. Chem. C.

[cit34] Mironova-Ulmane N., Kuzmin A., Steins I., Grabis J., Sildos I., Pärs M. (2007). J. Phys.: Conf. Ser..

[cit35] Wang W., Liu Y., Xu C., Zheng C., Wang G. (2002). Chem. Phys. Lett..

[cit36] Wang C., Zhao Y., Su D., Ding C., Wang L., Yan D., Li J., Jin H. (2017). Electrochim. Acta.

[cit37] Mironova-Ulmane N., Kuzmin A., Sildos I., Puust L., Grabis J. (2019). Latv. J. Phys. Tech. Sci..

[cit38] Bala N., Singh H. K., Verma S., Rath S. (2020). Phys. Rev. B: Condens. Matter.

[cit39] Marciuš M., Ristić M., Ivanda M., Musić S. (2012). J. Alloys Compd..

[cit40] Stern L.-A., Hu X. (2014). Faraday Discuss..

[cit41] Kuo D.-Y., Kawasaki J. K., Nelson J. N., Kloppenburg J., Hautier G., Shen K. M., Schlom D. G., Suntivich J. (2017). J. Am. Chem. Soc..

[cit42] Hong W. T., Welsch R. E., Shao-Horn Y. (2016). J. Phys. Chem. C.

[cit43] Li G., Anderson L., Chen Y., Pan M., Abel Chuang P.-Y. (2018). Sustain. Energy Fuels.

[cit44] Zhu K., Shi F., Zhu X., Yang W. (2020). Nano Energy.

[cit45] Wang Y.-H., Li L., Shi J., Xie M.-Y., Nie J., Huang G.-F., Li B., Hu W., Pan A., Huang W.-Q. (2023). Adv. Sci..

[cit46] Susman M. D., Pham H. N., Zhao X., West D. H., Chinta S., Bollini P., Datye A. K., Rimer J. D. (2020). Angew. Chem., Int. Ed..

[cit47] Xiao C., Lu B.-A., Xue P., Tian N., Zhou Z.-Y., Lin X., Lin W.-F., Sun S.-G. (2020). Joule.

[cit48] Ning Y., Guan Y., Zhang N., Song W., Zhang F., Chen L., Chai F. (2022). ChemistrySelect.

[cit49] Bilal M., Altaf A., Bint-E-Khalid E., Zafar H. K., Tahir N., Nafady A., Wahab M. A., Shah S. S. A., Najam T., Sohail M. (2023). RSC Adv..

[cit50] Sun T., Wang D., Mirkin M. V., Cheng H., Zheng J.-C., Richards R. M., Lin F., Xin H. L. (2019). Proc. Natl. Acad. Sci. U. S. A..

[cit51] Yu S., Xiong Z., Zhou H., Zhang Q., Wang Z., Ma F., Qu Z., Zhao Y., Chu X., Zhang X., You J. (2023). Science.

[cit52] Zhang B., Su J., Guo X., Zhou L., Lin Z., Feng L., Zhang J., Chang J., Hao Y. (2020). Adv. Sci..

[cit53] Bhanuchandar S., Vinothkumar G., Arunkumar P., Sribalaji M., Keshri A. K., Babu K. S. (2023). J. Mater. Sci..

[cit54] Molaei R., Bayati R., Narayan J. (2013). Cryst. Growth Des..

