# Supplementary material for: Morphology-driven oxygen evolution performance of NiOx nanostructures and implications for hole transport in perovskite solar cells
Source: RSC Adv. 2026 Feb 18;16(11):9796–805. doi: 10.1039/d6ra00607h (PMC12914240; doi:10.1039/d6ra00607h)
Supplement: RA-016-D6RA00607H-s001 [file RA-016-D6RA00607H-s001.pdf]

Supplementary Information (SI) for RSC Advances.
This journal is © The Royal Society of Chemistry 2026

Supplementary Information

Morphology-Driven Oxygen Evolution Performance of NiO_x Nanostructures and Implications for Hole Transport in Perovskite Solar Cells

Prabhu Bharathan,[a] Can Li,[b] Bipin Rijal,[c] Lihua Zhang,[d] Areesha Maryam,[b] Joseph Delgado,[b] Kim Kisslinger,[d] Adyasa Priyadarsini,[e] Mahesh Nepal,[c] Tanka P. Bhushal,[c] Tara P. Dhakal,*[a,c] Shyam Kattel*[f] and Jiye Fang*[a,b]

[a]Materials Science and Engineering Program, State University of New York at Binghamton, Binghamton, New York 13902, United States.

[b]Department of Chemistry, State University of New York at Binghamton, Binghamton, New York 13902, United States.

[c]Department of Electrical and Computer Engineering, Center for Autonomous Solar Power (CASP), Binghamton University, Binghamton, New York 13902, United States.

[d]Center for Functional Nanomaterials, Brookhaven National Laboratory, Upton, New York 11973, United States.

[e]Department of Physics, Florida A&M University, Tallahassee, Florida 32307, United States.

[f]Department of Physics, University of Central Florida, Orlando, Florida 32816, United States.

* Corresponding Authors.

e-mail addresses:

tdhakal@binghamton.edu (T. P. Dhakal);

shyam.kattel@ucf.edu (S. Kattel);

jfang@binghamton.edu (J. Fang).

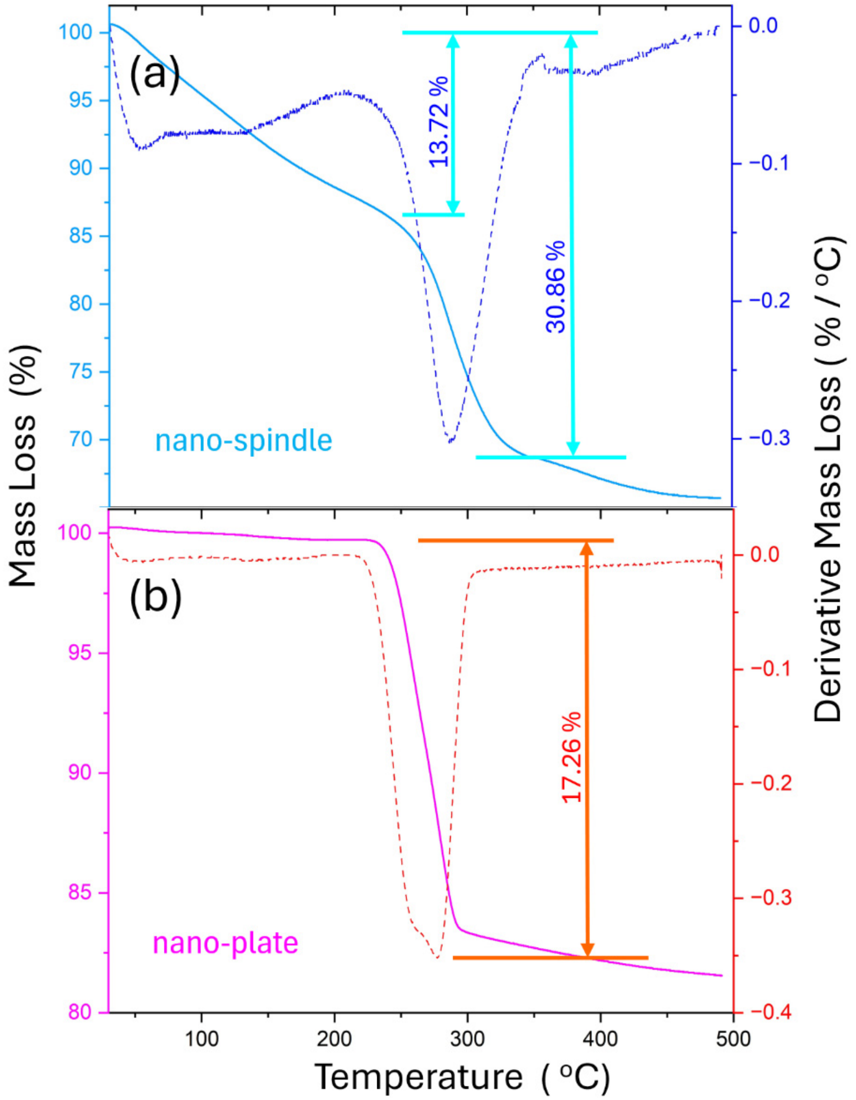

Fig. S1. Thermogravimetric analysis (TGA) and derivative thermogravimetry (DTG) plots, showing the thermal transformation of hydroxide-based (a) nano-spindles and (b) nano-plates, synthesized *via* Route (A) and Route (B), respectively.

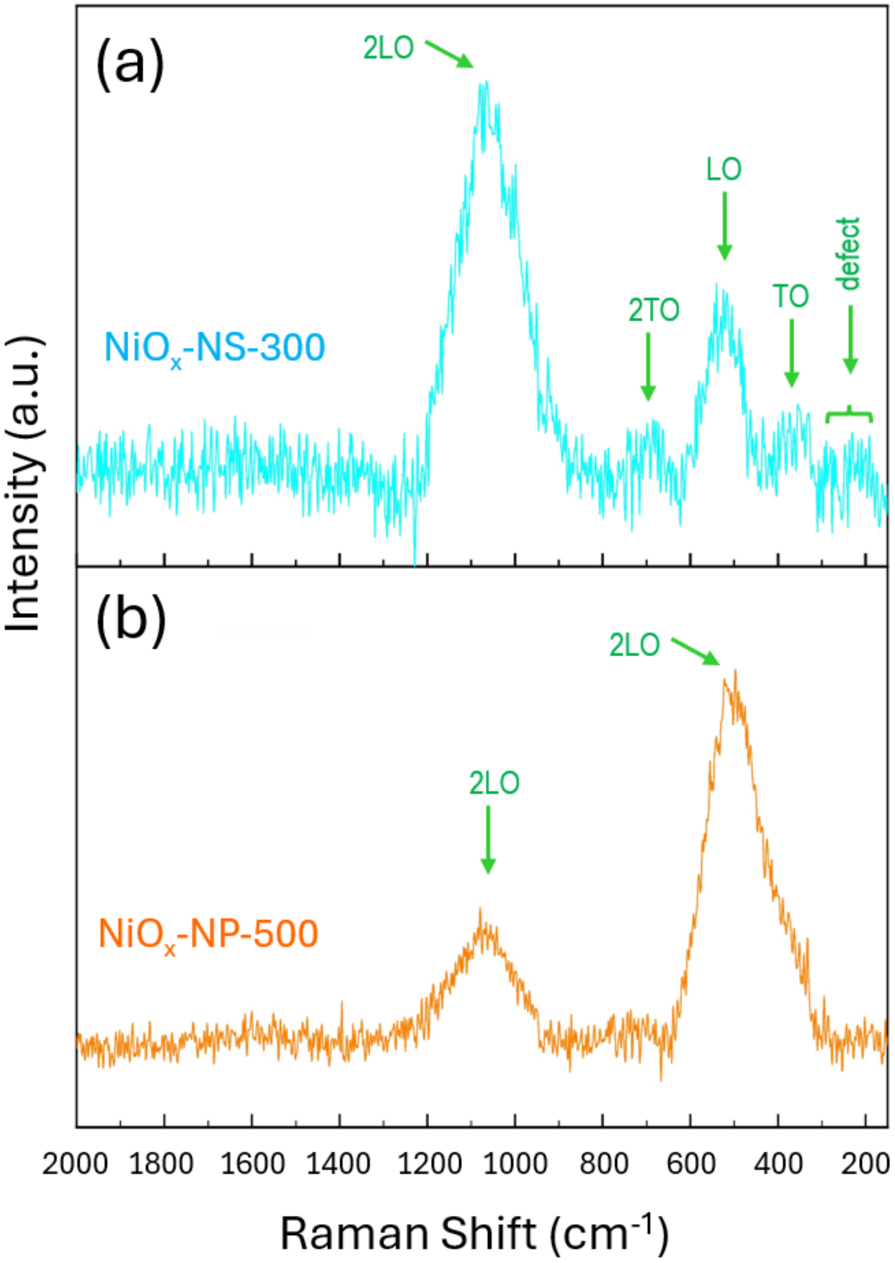

Fig. S2. Room temperature Raman spectra (λ_{ex} = 532 nm) of pristine NiO_x-NS-300 and NiO_x-NP-500.

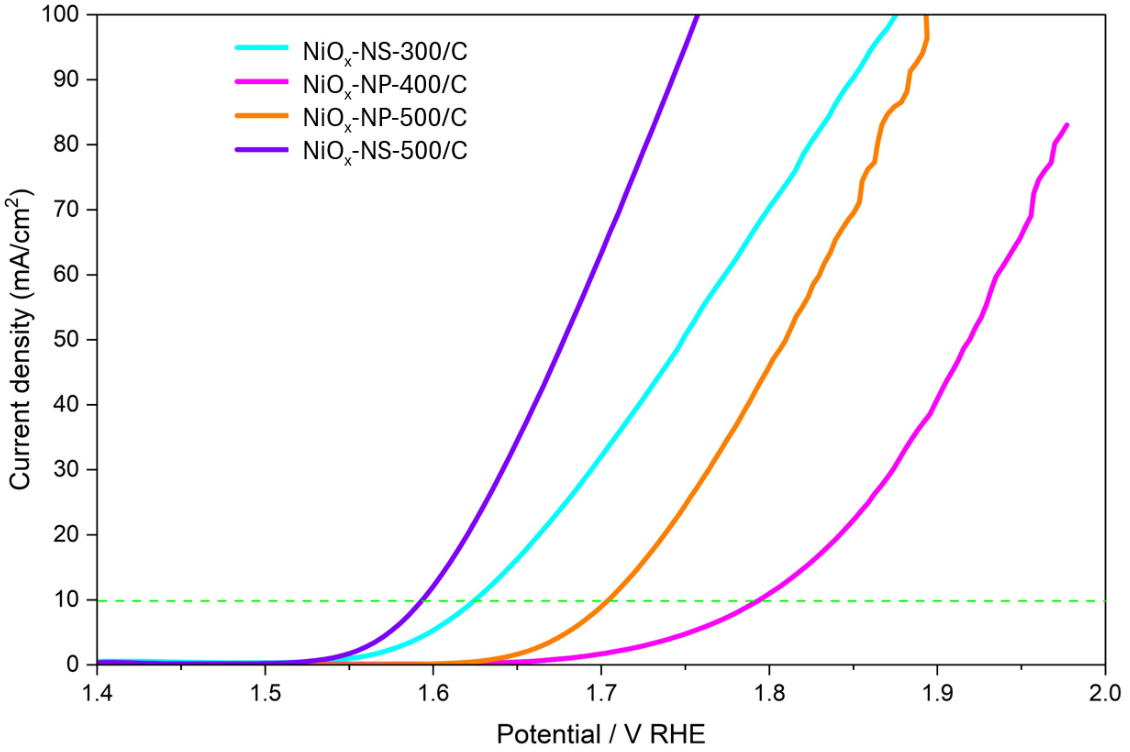

Fig. S3. Polarization curves (current density *vs*. potential) recorded in 1 M KOH at an electrode rotation speed of 1600 rpm. At benchmark current density of 10 mA/cm^2 (shown as the horizontal dashed line), OER overpotentials for NiO_x-NS-300/C, NiO_x-NP-400/C, NiO_x-NS-500/C, and NiO_x-NP-500/C are 395, 565, 364, and 474 mV, respectively.

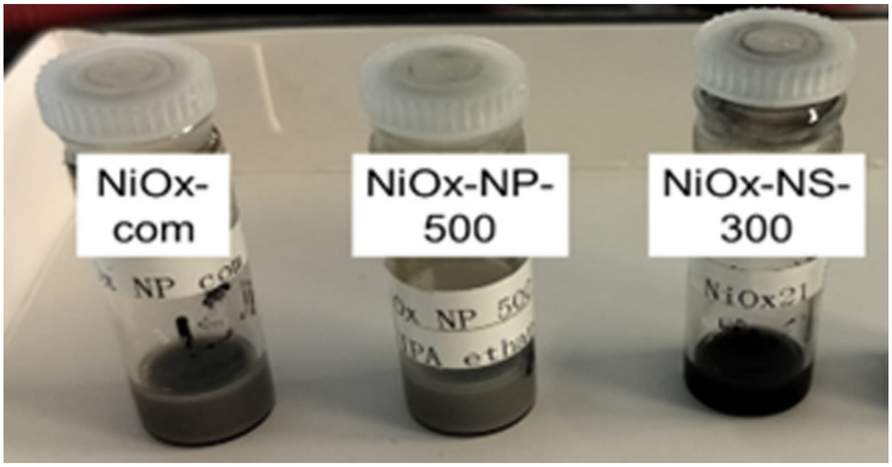

Fig. S4. Photographs of inks prepared from NiO_x-com (commercial sample), NiO_x-NP-500, and NiO_x-NS-300, showing distinct color differences.
